# Agreement and reliability of force-plate, smartphone-based, and wearable-sensor countermovement jump assessments for neuromuscular monitoring in male professional volleyball players

**DOI:** 10.3389/fphys.2026.1902583

**Published:** 2026-08-14

**Authors:** Bartosz Wilczyński, Tymon Środa, Mateusz Sikorski, Mariola Gepfert, Jakub Jarosz, Katarzyna Zorena

**Affiliations:** 1 Department of Immunobiology and Environmental Microbiology, Medical University of Gdansk, Gdańsk, Poland; 2 Gdansk Medical Academy of Applied Sciences, Gdansk, Poland; 3 Institute of Sport Sciences, Academy of Physical Education, Katowice, Poland

**Keywords:** athlete monitoring, exercise physiology, measurement error, neuromuscular performance, smartphone video, wearable sensor

## Abstract

**Background:**

Countermovement jump testing is widely used to infer lower-limb neuromuscular performance, readiness, fatigue, and adaptation in applied exercise physiology. However, commercially available force-plate, inertial-sensor, and smartphone-based systems use different signal sources and calculation methods, which may affect the physiological interpretation of jump-performance changes.

**Objective:**

To evaluate the agreement and test–retest reliability of bilateral and single-leg countermovement jump outcomes obtained from force plates, an inertial measurement unit, and a smartphone application in male volleyball players.

**Methods:**

Twenty professional male volleyball players were enrolled. Bilateral and left/right single-leg countermovement jump trials were recorded concurrently using VALD ForceDecks, MyJump Lab 3, and Baiobit. VALD impulse–momentum-derived jump height was used as the reference outcome. Agreement analyses compared MyJump Lab 3 and Baiobit with VALD using bias, limits of agreement, ICC(2,1), SEM, and MDC_95_. Test–retest reliability was assessed between two sessions separated by 12 days. Sensitivity analyses examined VALD flight-time-derived height, MyJump flight time, best-trial values, and strict quality-control exclusions.

**Results:**

MyJump Lab 3 systematically overestimated VALD impulse–momentum jump height by +1.88 to +4.16 cm. This discrepancy was markedly attenuated when VALD flight-time-derived height was used as the reference, while MyJump flight-time bias was negligible, indicating a calculation-method effect rather than temporal event-detection error. Baiobit showed smaller mean bias in some single-leg conditions but poorer individual-level agreement, with wider limits of agreement and less consistent absolute-agreement ICCs. VALD showed the strongest bilateral CMJ reliability (ICC = 0.929; MDC_95_ = 2.79 cm), whereas single-leg reliability was generally lower.

**Conclusion:**

Commercially available jump-testing systems can lead to method-dependent differences in the interpretation of neuromuscular performance. Device-, task-, and metric-specific measurement error should be considered before using jump-height changes to infer readiness, fatigue, or adaptation in professional male volleyball players.

## Introduction

Field-based neuromuscular testing is now routine in applied sport science, supporting athlete monitoring, performance profiling, and rehabilitation decision-making (Asimakidis et al., 2024; Taberner et al., 2025; Wilczyński et al., 2025). This trend is reinforced by the rapid growth of the sports-device market, projected to increase from USD 3.34 billion in 2022 to USD 8.91 billion by 2030 (Grand View Research, 2023). Portable force plates, inertial measurement units (IMU), and smartphone applications have substantially increased access to field-based jump testing outside laboratory settings. However, wider accessibility also creates a methodological challenge: measurements derived from different technologies may not be directly comparable because they differ in validity, reliability, signal acquisition, and outcome derivation (Bishop et al., 2017, 2022).

The countermovement jump (CMJ) is one of the most widely used field tests because it provides a rapid, low-fatigue estimate of lower-limb neuromuscular performance. It is used for athlete profiling, training-load monitoring, fatigue assessment, and return-to-sport decision-making (Claudino et al., 2017; Bishop et al., 2022; Osborne et al., 2025). This is particularly relevant in volleyball, where players are exposed to repeated high-intensity jumping actions. Recent volleyball-specific evidence indicates that high-intensity jump counts are more closely related to force-plate-derived neuromuscular performance metrics than total jump counts or session duration (Sanders et al., 2025), supporting the practical need for sensitive and repeatable jump-monitoring procedures in this sport.

Bilateral CMJ is the most common approach, but single-leg CMJ (SL-CMJ) variants provide limb-specific information on inter-limb differences and unilateral force-production capacity (Bishop et al., 2017; Donskov et al., 2021). In volleyball, this is practically relevant because jumping and landing demands are not always symmetrical, and repeated approach, block, and landing actions may expose athletes to side-to-side differences in force production and movement strategy. Longitudinal monitoring data from professional volleyball also suggest that CMJ testing procedures must be rigorous, because changes in jump execution can affect jump-height values and reduce or increase measurement variability (Masel and Maciejczyk, 2024). Beyond jump height, variables such as the flight-time-to-contraction-time ratio (FT: CT), modified reactive strength index (RSI-modified), and impulse offer complementary information on jump strategy and neuromuscular function (Bishop et al., 2022; McMahon et al., 2022), but only if they are reliably and consistently defined by the device used.

The main methodological issue is that different technologies do not estimate jump outcomes in the same way. Force plates derive outcomes from force–time data (Collings et al., 2024), IMU systems estimate movement from segmental acceleration and orientation signals (Villa et al., 2024), and smartphone applications commonly rely on video-derived flight time or image-processing methods (Balsalobre-Fernández and Varela-Olalla, 2024). Even within force-plate testing, jump-height estimates can differ depending on whether impulse–momentum or flight-time-based calculations are used (Eythorsdottir et al., 2024). Recent studies have reported acceptable validity or reliability for individual smartphone applications, IMU-based devices, and portable jump-testing tools (Balsalobre-Fernández and Varela-Olalla, 2024; Puljić et al., 2024; Cameron et al., 2025). However, device-specific validity does not establish practical equivalence between systems. Outputs from different systems should not be compared directly unless agreement and reliability have been evaluated under the same task and field-testing conditions.

Therefore, this study aimed to evaluate the agreement and test–retest reliability of bilateral and single-leg CMJ outcomes obtained from force plates, an IMU system, and a smartphone application in male volleyball players from a professional club setting. We examined whether jump-height outputs from MyJump Lab 3 and Baiobit showed sufficient agreement with VALD ForceDecks impulse-momentum-derived jump height to support practical method-to-method comparison under applied field-testing conditions. Secondary analyses examined available temporal, force-time, and strategy-related variables. Consistent with the preregistration, we hypothesised systematic device differences for jump height, with bias varying by device and jump condition. We expected disagreement to be larger for the IMU-based system and for single-leg CMJ conditions because these outcomes are more dependent on segmental sensor behavior, unilateral movement control, and task execution variability. The main contribution of this study is the same-trial comparison of force-plate, smartphone-video, and IMU-derived CMJ outputs across bilateral and single-leg tasks in a professional volleyball environment, with interpretation centered on individual-level agreement error and measurement precision.

## Methods

### Participants

Twenty male professional volleyball players were enrolled (age: 17.85 ± 0.85 years; body mass: 86.09 ± 8.67 kg; height: 195.20 ± 5.13 cm). At the time of testing, all players held formal player contracts and were affiliated with a professional volleyball club structure (Trefl Gdańsk, Poland). They competed primarily in Polish II- and III-level league competitions, with some players periodically involved in the senior top-tier professional environment.

Consistent with standardization procedures used in volleyball performance-testing research, participants were asked to maintain their usual dietary and training routines before each testing session and to avoid any new ergogenic supplements, stimulants, or unusual recovery strategies during the preceding week (Gepfert et al., 2026). Regular habitual supplementation, if already used, was not modified. Players were eligible if they were active club players and had no acute musculoskeletal injury at the time of testing.

All participants were informed about the study procedures before testing. Written informed consent was obtained from adult participants. For participants under 18 years of age, written consent was obtained from a parent or legal guardian, and the underage participant provided assent before participation. The study complied with the Declaration of Helsinki, was approved by the Bioethics Committee for Scientific Research at the Medical University of Gdańsk, Poland (approval number: KB/30/2026; approval date: 20 February 2026), and was preregistered on OSF (10.17605/OSF.IO/QTB6C).

### Sample-size rationale

No formal *a priori* sample-size or power calculation was performed. The study used pragmatic complete-roster sampling because data collection was embedded within in-season field testing at a single professional volleyball club. All eligible and available players were invited, resulting in 20 enrolled athletes. The preregistration did not specify a predetermined sample-size target; therefore, the results were interpreted using an estimation-focused framework, with device- and task-specific effective sample sizes, confidence intervals, SEM, MDC_95_, and limits of agreement used to characterize measurement uncertainty.

### Participant flow, missing data, and effective analysis samples

Data availability and eligibility were assessed separately for each participant, session, device, jump condition, and outcome. Of the 20 enrolled players, 13 contributed at least one eligible Session 1 or Session 2 record, 11 contributed at least one matched Session 1–Session 2 reliability pair, and nine had complete matched jump-height records across all nine device–jump combinations. The main documented reasons for unavailable or incomplete matched data were one pain-related Session 2 non-completion, two follow-up absences for which the reasons were undocumented, and device-, acquisition-, export-, or quality-control-specific data losses affecting four participants.

Participants with incomplete records were not excluded from all analyses and contributed whenever eligible data were available. No missing observations were imputed. Agreement analyses required concurrent eligible measurements from the relevant device pair. Test–retest reliability analyses required valid matched Session 1 and Session 2 measurements for the relevant device and jump condition. Consequently, effective sample sizes differed across analyses and are reported separately in the main tables and **Supplementary Table 4**. Primary reliability analyses included 10–11 paired participants, whereas strict-QC sensitivity analyses included 9–11 paired participants.

### Study design and setting

Testing was conducted in the same indoor gymnasium at the Trefl Gdańsk training facility, in the afternoon and before the players’ main motor training session. Testing was scheduled at a similar time of day across sessions to minimize potential diurnal variation in neuromuscular performance. Three systems recorded the same jump trials concurrently: VALD ForceDecks, MyJump Lab 3, and Baiobit, representing force-plate, smartphone-video, and inertial measurement technologies, respectively.

Data were collected across four acquisition waves during the competitive season: a pilot session on 24 February 2026, Session 1 on 19 March 2026, Session 2 on 31 March 2026, and Session 3 on 2 April 2026. Sessions 1 and 2 formed the primary analysis set and were separated by 12 days. Testing was scheduled in coordination with the club staff and conducted before the main training session. Players continued their usual team training and match-related routines between testing sessions; these activities were not experimentally controlled but remained part of the normal in-season training environment. Training load on the day preceding testing was not experimentally standardized or recorded. The pilot session was used to refine the field workflow and was retained only for predefined exploratory sensitivity analyses when data quality criteria were met; Session 3 was also treated as exploratory. Reporting followed the Guidelines for Reporting Reliability and Agreement Studies (GRRAS), and the completed checklist is provided in the Supplementary Material.

### Measurement systems

VALD ForceDecks (VALD Performance, Brisbane, Australia) is a dual force-plate system that records ground reaction force at 1000 Hz. Jump height was derived as the primary reference outcome using the impulse–momentum method, based on take-off velocity calculated from the vertical force–time signal. VALD flight-time-derived jump height was retained for sensitivity analyses.

MyJump Lab 3 (Carlos Balsalobre-Fernández, Spain) was installed on an iPhone 16 (Apple Inc., Cupertino, CA, USA). Videos were recorded at 240 fps. The smartphone was mounted on a tripod 150 cm from the participant and 70 cm above the floor to maintain full-body visibility throughout take-off, flight, and landing. Take-off and landing frames were manually identified within the application by one trained evaluator. The same evaluator processed all videos to maintain consistency across participants, sessions, and jump conditions. Formal blinding to the outputs of the other devices was not implemented; however, frame selection was performed from the video recordings and did not use VALD ForceDecks or Baiobit outputs. Before formal data extraction, the evaluator completed a familiarization and practice-scoring phase using tutorial and pilot recordings, including repeated frame-identification exercises to standardize the procedure. These practice checks were used for training and procedural calibration only and were not retained as a formal intra-rater reliability dataset. Inter-rater reliability was not applicable because a second independent evaluator did not score the recordings, and formal intra-rater reliability statistics were not calculated. Jump height was derived from flight time using h = g × t^2^/8.

Baiobit (Rivelo Srl—BTS Bioengineering Group, Milan, Italy) is a wearable inertial measurement unit incorporating accelerometer, gyroscope, and magnetometer signals. The sensor was positioned over the lower lumbar region at L4–L5 and secured in standing using the manufacturer-supplied elastic strap. The strap was fastened firmly while the participant was standing relaxed, and the sensor position and strap tension were checked visually before each testing block. This procedure was intended to minimize relative sensor movement during jumping, but sensor–skin displacement was not measured directly. Jump height and secondary metrics were exported from the manufacturer’s proprietary software.

Available technical details for all measurement systems are provided in **Supplementary Table 5**.

### Countermovement jump protocol

Participants performed all jumps on the force plates with hands on hips to standardize upper-limb contribution and support consistent video-based event detection. Each session began with a standardized warm-up consisting of two practice bilateral countermovement jumps (CMJ) and two practice single-leg countermovement jumps (SL-CMJ) on each leg, followed by three minutes of rest. The task order was fixed and non-randomized for all participants and sessions to reflect the applied club-monitoring workflow and to maintain identical task sequencing across participants and sessions. Testing was completed in the following order: three bilateral CMJ trials, three minutes of rest, three left SL-CMJ trials, three minutes of rest, and three right SL-CMJ trials. Passive rest between repeated trials was 30 seconds. All trials were supervised by the same researcher using standardized verbal instructions and demonstration. Participants were instructed to perform a rapid countermovement, transition immediately into the upward phase, and “jump as high as possible.” Verbal encouragement was provided to promote maximal effort across trials.

For SL-CMJ trials, participants jumped from and landed on the tested limb. The non-tested limb was kept flexed at the knee and was not allowed to contact the tested limb or the force plates. Swinging of the non-tested limb was not permitted. Trial execution and validity criteria were standardized in line with previous vertical jump testing procedures (Impellizzeri et al., 2007; Spudić et al., 2025). Trials were repeated if the participant used an incorrect technique, lost balance on landing, contacted the ground with the non-tested limb, removed the hands from the hips, or if a device-recording error occurred.

### Data processing and quality control

Manufacturer-exported data from all three systems were harmonized into a long-format dataset, with each row representing one participant × session × jump type × device combination. Trial-level data were aggregated using the mean of valid trials as the primary analytical value. The best valid trial was retained for sensitivity analyses. Quality-control decisions were made separately for each participant, session, device, jump condition, and outcome before statistical modelling.

Records were classified as: (1) QC-clean and eligible; (2) QC-flagged but eligible; (3) excluded for a specific variable; or (4) excluded at the complete record level. A flagged record was retained when the relevant acquisition and matched trial were available, the outcome remained technically interpretable, and the documented concern did not invalidate the comparison under analysis. Complete records were excluded when the relevant acquisition was missing or incomplete, the trial did not satisfy the predefined execution criteria, recordings could not be matched reliably across devices, or a technical or processing artefact directly compromised the analyzed outcome. When an artefact affected only one derived variable, that variable was excluded while the remaining eligible outcomes from the same record were retained.

The primary analyses included both QC-clean records and eligible QC-flagged records. Strict-QC sensitivity analyses included QC-clean records only. No missing values were imputed. Large inter-device differences were not classified as outliers because such differences represented the measurement-agreement question under investigation. The complete participant-level audit trail and diagnostic logs are available in the OSF repository.

### Statistical analysis

The primary analysis examined jump-height agreement between MyJump Lab 3 and VALD, and between Baiobit and VALD, separately for bilateral CMJ, left SL-CMJ, and right SL-CMJ. VALD impulse–momentum jump height was used as the reference because it is derived from vertical force–time data and take-off velocity rather than flight time alone. Bias was calculated as comparator device minus VALD.

For primary device-agreement analyses, eligible Session 1 and Session 2 observations were pooled at the participant-session level. This approach was used because the agreement question concerned device-to-reference differences across applied monitoring occasions, and each participant-session observation represented a distinct field-testing occasion in which all devices recorded the same trials concurrently. Because some athletes contributed observations from both sessions, supplementary sensitivity analyses examined the influence of repeated observations within participants. First, participant-level aggregated Bland–Altman analyses were conducted by averaging available Session 1 and Session 2 differences within each athlete before estimating bias and 95% limits of agreement. Second, repeated-measures Bland–Altman analyses were conducted using mixed-effects models in which the device difference was modelled with athlete as a random effect. Repeated-measures limits of agreement were estimated from the fixed intercept and the combined between-athlete and residual variance components.

Agreement was quantified using Bland–Altman mean bias, 95% confidence intervals for bias, 95% limits of agreement, ICC(2,1), SEM, and MDC_95_. ICC(2,1) was specified as a two-way random-effects, absolute-agreement, single-measure model and is reported with 95% confidence intervals. Confidence intervals for Bland–Altman limits of agreement were estimated in supplementary analyses using participant-level cluster bootstrap with 5000 resamples to account for repeated participant-session observations.

Proportional bias was assessed by regressing the device difference on the pairwise mean. As an additional check for magnitude-dependent error variance, absolute differences were inspected visually and regressed against the pairwise mean. Absolute limits of agreement were retained because these checks did not provide clear evidence of heteroscedasticity in the primary agreement analyses, although the limited effective sample size was recognized as reducing the sensitivity of these assessments.

To provide practical context, the smallest worthwhile change (SWC) was estimated descriptively as 0.2 × the baseline between-athlete SD using VALD Session 1 values. SWC was treated as an exploratory benchmark and compared with SEM and MDC_95_ values. No *a priori* clinically acceptable agreement threshold was specified, and formal equivalence testing was not performed. Device comparisons were therefore interpreted descriptively using bias, 95% limits of agreement, ICC(2,1) with confidence intervals, SEM, MDC_95_, and exploratory SWC benchmarks. To complement the absolute Bland–Altman analyses in centimeters, relative-error Bland–Altman analyses were conducted as sensitivity analyses using percentage differences calculated as 100 × (comparator − VALD)/[(comparator + VALD)/2]. No percentage-based acceptability bands were imposed because no such thresholds were prespecified.

Secondary agreement analyses compared MyJump Lab 3 and VALD flight time to examine whether height differences reflected temporal event detection or the height-calculation method. VALD and Baiobit peak-power outputs were compared descriptively as an exploratory manufacturer-output comparison because the devices use non-equivalent peak-power algorithms. Sensitivity analyses examined VALD flight-time-derived height as the reference, best-trial values, temporal stability of device bias across acquisition waves, strict-QC analyses excluding retained flagged records, and participant-level bias profiles relative to VALD.

Within-device test–retest reliability was assessed from Session 1 to Session 2 for jump height as the primary outcome. Reliability was quantified using Bland–Altman bias and limits of agreement, ICC(2,1), SEM, MDC_95_, and CV%. Bias was calculated as Session 2 minus Session 1. SEM was estimated from the residual error term of the two-way absolute-agreement model. MDC_95_ was calculated as SEM × 1.96 × √2. CV% was calculated as (SD_diff/√2)/grand mean × 100. Secondary reliability outcomes were summarized in the Supplementary Material, with full device-, outcome-, and jump-specific estimates archived on OSF. Analyses were performed in Python 3.13. Interpretation focused on estimation precision rather than null-hypothesis testing.

## Results

### Data availability and quality control

The final three-device source dataset contained 342 participant × session × jump × device rows across all acquisition waves. After predefined attendance, pain-related, device-specific, acquisition, export, and variable-level quality-control decisions, 332 records remained eligible for analysis, including 309 QC-clean records and 23 eligible QC-flagged records. Eligible QC-flagged records were retained in the primary analyses and excluded in strict-QC sensitivity analyses.

Of the 20 enrolled players, 13 contributed at least one eligible Session 1 or Session 2 record, and 11 contributed at least one matched Session 1–Session 2 reliability pair. Primary agreement analyses included 21–24 participant-session observations from 12–13 participants, depending on the device–jump comparison. Primary test–retest reliability analyses included 10–11 paired participants, depending on the device and jump condition. Device- and jump-specific sample sizes are reported in **Tables 1** and **2**, and the participant-flow summary is provided in **Supplementary Table 4**. Full quality-control audit trails, data-coverage summaries, and diagnostic logs are archived on OSF.

**Table 1:** Primary agreement of jump-height estimates against VALD impulse–momentum-derived jump height.

Jump	Device comparison	n observations/participants	Bias (cm)	95% LoA (cm)	ICC(2,1)	95% CI	SEM (cm)	MDC_95_ (cm)
CMJ	MyJump Lab 3 vs VALD	24/13	4.16	−1.24 to 9.56	0.54	[−0.10, 0.84]	1.95	5.40
CMJ	Baiobit vs VALD	22/12	−2.59	−10.92 to 5.74	0.49	[0.09, 0.75]	3.00	8.33
SL-CMJ-L	MyJump Lab 3 vs VALD	23/13	1.88	−2.23 to 6.00	0.84	[0.36, 0.94]	1.48	4.11
SL-CMJ-L	Baiobit vs VALD	21/12	−0.46	−5.83 to 4.90	0.81	[0.59, 0.92]	1.94	5.37
SL-CMJ-R	MyJump Lab 3 vs VALD	23/13	2.36	−2.16 to 6.89	0.55	[−0.02, 0.82]	1.63	4.53
SL-CMJ-R	Baiobit vs VALD	21/12	0.74	−7.42 to 8.89	0.20	[−0.25, 0.58]	2.94	8.16

Values are based on the mean of valid trials, with Sessions 1 and 2 pooled at the participant-session level. Bias was calculated as comparator − VALD; positive values indicate higher estimates from the comparator device. VALD impulse–momentum-derived jump height was used as the reference. ICC(2,1) was specified as a two-way random-effects, absolute-agreement, single-measure model. SEM and MDC_95_ are reported as agreement-error indices for device-comparison analyses. Cluster-bootstrap confidence intervals for the limits of agreement are provided in **Supplementary Table 6**. LoA, limits of agreement; ICC, intraclass correlation coefficient; SEM, standard error of measurement; MDC_95_, minimal detectable change at 95% confidence; CMJ, countermovement jump; SL-CMJ, single-leg countermovement jump.

**Table 2:** Test–retest reliability of jump-height estimates from session 1 to session 2.

Device	Jump	n	Bias S2 − S1 (cm)	95% LoA (cm)	ICC(2,1)	95% CI	SEM (cm)	MDC_95_ (cm)	CV%
VALD	CMJ	11	−0.38	−3.17 to 2.42	0.93	[0.77, 0.98]	1.01	2.79	2.5
VALD	SL-CMJ-L	10	0.54	−5.51 to 6.59	0.82	[0.44, 0.95]	2.18	6.05	11.1
VALD	SL-CMJ-R	10	0.21	−5.64 to 6.05	0.56	[−0.11, 0.87]	2.11	5.84	11.3
MyJump Lab 3	CMJ	11	1.17	−2.69 to 5.03	0.90	[0.64, 0.97]	1.39	3.86	3.1
MyJump Lab 3	SL-CMJ-L	11	−0.29	−3.11 to 2.52	0.95	[0.84, 0.99]	1.02	2.82	4.7
MyJump Lab 3	SL-CMJ-R	11	−0.40	−3.79 to 3.00	0.78	[0.39, 0.94]	1.23	3.40	5.7
Baiobit	CMJ	10	−2.01	−10.64 to 6.62	0.51	[−0.06, 0.85]	3.11	8.63	8.3
Baiobit	SL-CMJ-L	10	−1.15	−8.16 to 5.86	0.57	[−0.02, 0.87]	2.53	7.01	13.3
Baiobit	SL-CMJ-R	10	0.56	−7.86 to 8.98	0.07	[−0.64, 0.66]	3.04	8.42	15.8

Jump-height values, bias, LoA, SEM, and MDC_95_ are reported in centimeters; CV is reported as percentage. Bias was calculated as Session 2 − Session 1; positive values indicate higher values at Session 2. ICC(2,1) was specified as a two-way random-effects, absolute-agreement, single-measure model. The primary reliability analysis included both QC-clean and eligible QC-flagged records; strict-QC sensitivity analyses are reported separately in the **Supplementary Material**. LoA, limits of agreement; ICC, intraclass correlation coefficient; SEM, standard error of measurement; MDC_95_, minimal detectable change at 95% confidence; CV%, coefficient of variation; CMJ, countermovement jump; SL-CMJ, single-leg countermovement jump.

### Primary agreement: jump height

**Figure 1:**
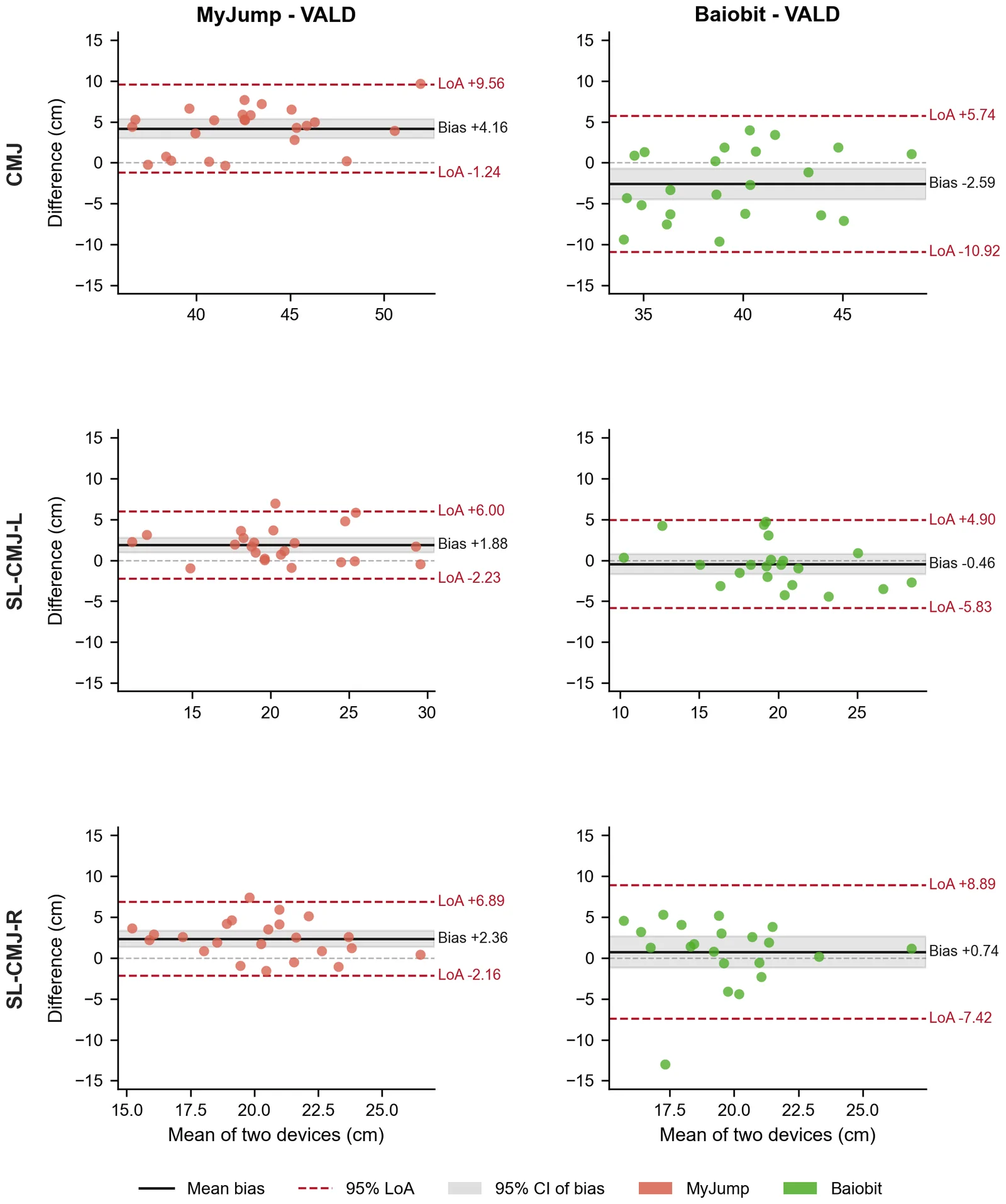
Bland–Altman plots for primary jump-height agreement against VALD impulse–momentum height. Rows represent jump conditions and columns represent device comparisons against VALD impulse–momentum jump height. Absolute Bland–Altman plots are shown in centimeters to preserve direct practical interpretability. Cluster-bootstrap confidence intervals for the limits of agreement are reported in **Supplementary Table 6**, and relative-error Bland–Altman plots are provided in **Supplementary Figure 5**. No predefined acceptability bands were imposed. The y-axis shows the device difference, calculated as comparator − VALD. The black solid line indicates mean bias, red dashed lines indicate 95% limits of agreement, and grey shading indicates the 95% confidence interval for the mean bias. CMJ, countermovement jump; SL-CMJ-L, left single-leg countermovement jump; SL-CMJ-R, right single-leg countermovement jump.

MyJump Lab 3 overestimated VALD impulse–momentum jump height in all jump conditions, with mean bias ranging from +1.88 to +4.16 cm (**Table 1**). The largest bias occurred in bilateral CMJ (+4.16 cm; 95% CI: +3.00 to +5.33), with 95% limits of agreement from −1.24 to +9.56 cm (**Figure 1**). Cluster-bootstrap confidence intervals for the limits of agreement are provided in **Supplementary Table 6**, and relative-error Bland–Altman plots are provided in **Supplementary Figure 5**. Absolute-agreement ICCs ranged from 0.54 to 0.84, and MDC_95_ values ranged from 4.11 to 5.40 cm.

Baiobit showed smaller mean bias than MyJump Lab 3 in both single-leg conditions, but wider limits of agreement across all jump conditions. For bilateral CMJ, Baiobit underestimated VALD by −2.59 cm, with 95% limits of agreement from −10.92 to +5.74 cm. Across jump conditions, Baiobit absolute-agreement ICCs ranged from 0.20 to 0.81 and MDC_95_ values ranged from 5.37 to 8.33 cm. The largest agreement error occurred in right SL-CMJ, where the limits-of-agreement width was 16.31 cm and the ICC was 0.20 (95% CI: −0.25 to 0.58).

### MyJump flight-time sensitivity

**Figure 2:**
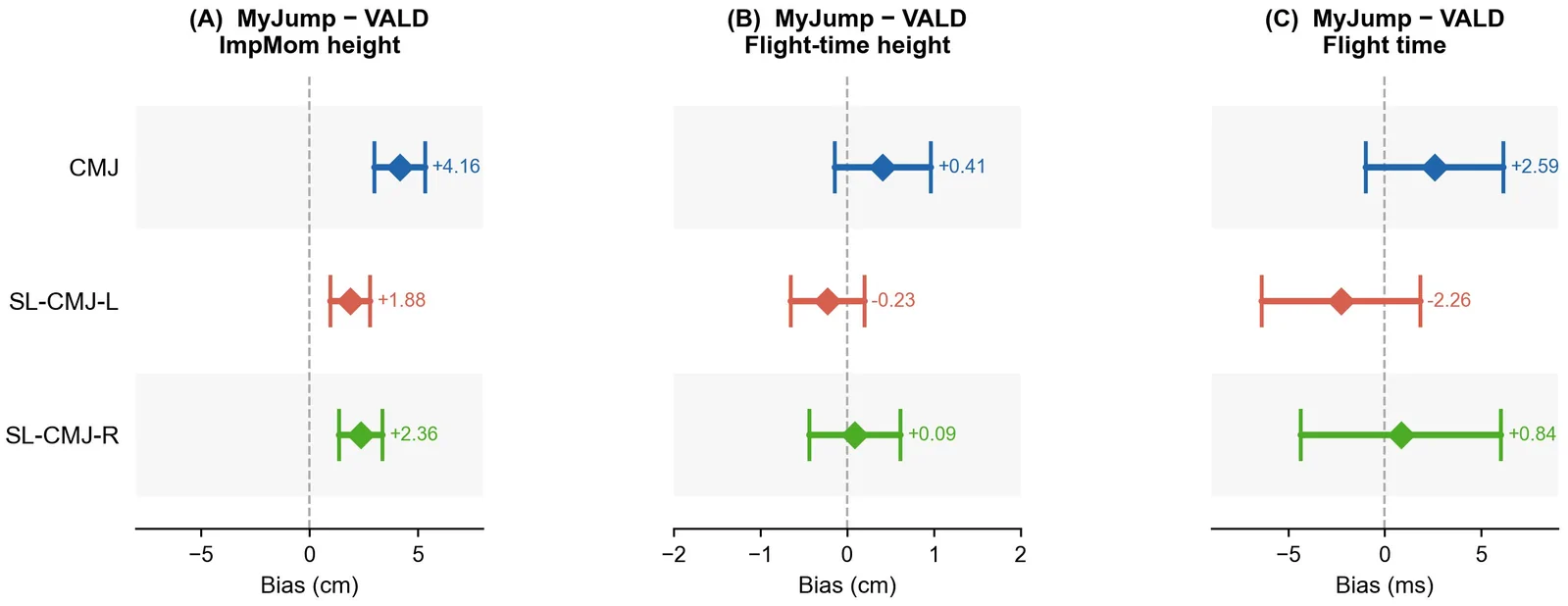
Mechanistic sensitivity analysis of MyJump Lab 3 bias. Panel **(A)** shows mean bias and 95% confidence intervals for MyJump Lab 3 jump height relative to VALD impulse–momentum-derived height. Panel **(B)** shows MyJump Lab 3 jump height relative to VALD flight-time-derived height. Panel **(C)** shows MyJump Lab 3 flight time relative to VALD flight time. Bias was calculated as MyJump Lab 3 minus VALD. The vertical dashed line indicates zero bias. The attenuation of jump-height bias when VALD flight-time-derived height was used as the reference supports the interpretation that the primary MyJump–VALD discrepancy was driven mainly by height-calculation method rather than temporal event detection.

MyJump Lab 3 showed negligible flight-time bias relative to VALD across all jump conditions (**Figure 2**; **Supplementary Table 2**), ranging from −2.3 to +2.6 ms, with high absolute-agreement ICCs (0.90–0.98). However, MyJump Lab 3 showed systematic positive jump-height bias when compared with VALD impulse–momentum height. When VALD flight-time-derived height was used as the reference, this bias was reduced to near zero: +0.41 cm for CMJ, −0.23 cm for SL-CMJ-L, and +0.09 cm for SL-CMJ-R.

### Test–retest reliability

**Figure 3:**
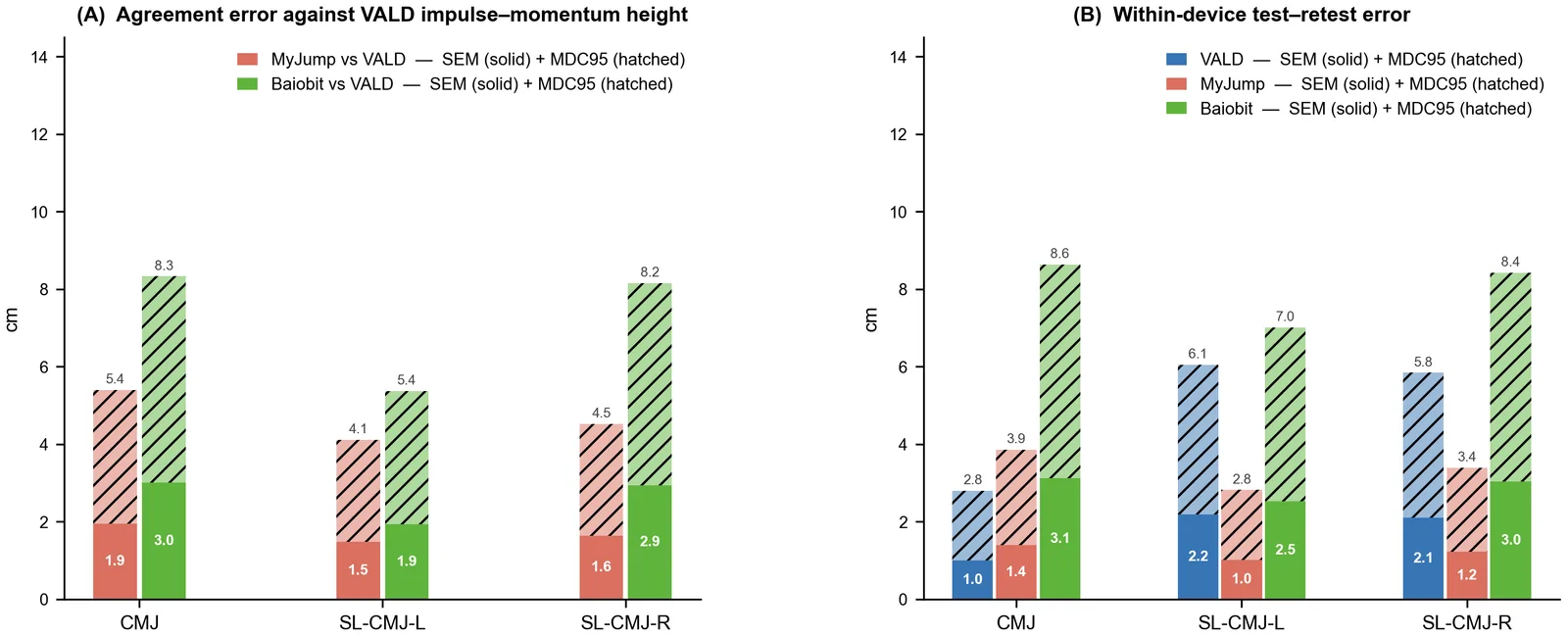
Measurement error for device agreement and test–retest reliability. Panel **(A)** shows SEM and MDC_95_ from device-comparison analyses against VALD impulse–momentum jump height. Panel **(B)** shows within-device test–retest SEM and MDC_95_ from Session 1 to Session 2. Each bar represents one device–jump condition. The solid lower segment represents SEM; the full bar height represents MDC_95_. Values inside bars show SEM, and values above bars show MDC_95_. Lower values indicate smaller individual-level measurement error.

VALD showed the strongest test–retest reliability for bilateral CMJ jump height (ICC = 0.93; MDC_95_ = 2.79 cm), whereas reliability was lower for single-leg conditions, particularly right SL-CMJ (ICC = 0.56; MDC_95_ = 5.84 cm) (**Table 2**). MyJump Lab 3 showed higher and more consistent reliability than Baiobit across jump types, with ICCs ranging from 0.78 to 0.95 and MDC_95_ values from 2.82 to 3.86 cm. Baiobit showed lower and less precise reliability estimates (ICC = 0.07–0.57), with larger MDC_95_ values (7.01–8.63 cm). Secondary test–retest reliability outcomes are summarized in **Supplementary Table 3**, with Bland–Altman plots for test–retest jump-height reliability provided in **Supplementary Figure 3** and ICC(2,1) patterns for secondary reliability outcomes visualized in **Supplementary Figure 4**. **Figure 3** summarizes the individual-level measurement error. Baiobit showed the largest MDC_95_ values for both device agreement and test–retest reliability, whereas MyJump Lab 3 showed lower test–retest MDC_95_ values across jump conditions.

### Sensitivity and exploratory analyses

Strict-QC sensitivity analyses restricted to QC-clean records preserved the direction and substantive interpretation of the primary agreement and reliability findings, indicating that retention of eligible QC-flagged records did not drive the principal conclusions (**Supplementary Table 7**). Best-trial sensitivity analyses showed the same directional pattern as the primary mean-of-trials analyses (**Supplementary Table 8**). MyJump Lab 3 continued to show systematic positive bias relative to VALD impulse–momentum-derived jump height, whereas Baiobit showed wider agreement error and less stable estimates, particularly in single-leg conditions.

The internal VALD comparison showed that flight-time-derived height exceeded impulse–momentum-derived height by +3.75 cm for CMJ, + 2.11 cm for SL-CMJ-L, and +2.28 cm for SL-CMJ-R. Secondary agreement analyses showed negligible MyJump Lab 3 flight-time bias relative to VALD and high absolute-agreement ICCs, whereas Baiobit peak-power outputs were substantially lower than VALD outputs and showed near-zero absolute-agreement ICCs. These findings support the interpretation that MyJump–VALD jump-height differences mainly reflected the height-calculation method, while Baiobit peak-power values should be treated as non-equivalent manufacturer-derived outputs.

Participant-level bias profiles showed visible between-athlete heterogeneity in device differences, particularly for Baiobit, indicating that group-level mean bias alone was insufficient for evaluating individual-level agreement (**Supplementary Figure 1**). Temporal-bias analyses suggested that device differences varied across available testing waves, but these estimates were based on unequal and sometimes small session-specific samples and should therefore be interpreted as exploratory checks rather than definitive evidence of device drift (**Supplementary Table 9**; **Supplementary Figure 2**).

Secondary reliability analyses showed excellent VALD reliability for bilateral CMJ force-time and strategy-related variables, including peak power, concentric impulse, RSI-modified, and flight time (ICC = 0.895–0.984). Reliability was generally lower for single-leg, landing-related, and Baiobit-derived secondary outcomes. MyJump Lab 3 flight-time reliability followed the same pattern as MyJump Lab 3 jump-height reliability, whereas Baiobit rate of force development and reactivity-related variables were unstable, particularly in single-leg conditions (**Supplementary Table 3**).

## Discussion

The main finding of this study is that commercially available systems used to assess CMJ performance can produce method-dependent, non-equivalent outputs during the same field-based exercise task. This has direct implications for exercise physiology because CMJ-derived changes are commonly interpreted as indicators of neuromuscular readiness, fatigue, and adaptation. MyJump Lab 3 systematically overestimated VALD impulse–momentum-derived jump height, whereas Baiobit showed smaller mean bias in some single-leg conditions but wider limits of agreement and poorer individual-level agreement. Therefore, small apparent changes in jump height may reflect device- or calculation-method effects rather than true physiological change.

The findings support a cautious interpretation of CMJ-derived neuromuscular monitoring data. In applied settings, jump-height changes are often used to infer fatigue, readiness, or adaptation, and recent studies have similarly treated CMJ-based performance testing as a practical marker of neuromuscular fatigue, recovery, and field-based athlete status (Deely et al., 2022; Pojskić et al., 2025). However, the present results show that the magnitude of device-related bias and MDC_95_ can equal or exceed changes that practitioners might otherwise interpret as meaningful. This is particularly important when athletes are monitored across congested training and competition periods, when expected physiological changes may be small. A monitoring system should therefore be treated as a method-specific measurement pathway rather than a generic representation of lower-limb neuromuscular status.

The MyJump findings indicate a calculation-method effect rather than poor event detection. MyJump Lab 3 showed negligible flight-time bias relative to VALD and high absolute-agreement ICCs for flight time, yet systematically overestimated VALD impulse–momentum jump height. This agrees with previous work supporting MyJump for flight-time-derived jump assessment and with evidence that jump-height values differ between flight-time and impulse–momentum methods (Balsalobre-Fernández et al., 2015; Bishop et al., 2022; Balsalobre-Fernández and Varela-Olalla, 2024; Eythorsdottir et al., 2024). MyJump Lab 3 may therefore be useful for within-device field monitoring, but its jump-height output should not be interpreted as equivalent to VALD impulse–momentum height.

The Baiobit results were less favorable for individual-level monitoring. Although mean bias was small in the single-leg conditions, this did not translate into sufficient individual-level agreement because limits of agreement were wide and absolute-agreement ICCs were low or inconsistent, particularly for right SL-CMJ. This partly differs from previous Baiobit research reporting good agreement for bilateral and single-leg CMJ height in athletes (Camuncoli et al., 2022). The discrepancy may reflect differences in the reference method, testing context, sample characteristics, and the present focus on individual-level agreement error rather than group-level association. Baiobit may therefore be useful for within-device longitudinal tracking, but its jump-height outputs should not be combined or compared directly without method-specific interpretation with force-plate impulse–momentum outputs when small individual changes are practically meaningful.

The reliability findings support a task- and metric-specific approach to CMJ monitoring. Bilateral CMJ was generally more stable than single-leg CMJ, particularly for VALD and Baiobit, whereas single-leg conditions showed larger MDC_95_ values and wider limits of agreement. This does not make SL-CMJ unsuitable. Previous studies have reported good-to-excellent reliability for unilateral jump tests, including SL-CMJ, in youth team-sport athletes, but also emphasised that reliability should be established within the target population and protocol (McCubbine et al., 2018; Donskov et al., 2021). In the present study, larger SL-CMJ MDC_95_ values indicate that small changes should not be interpreted without device- and task-specific error estimates. Secondary analyses extended this point beyond jump height: VALD showed excellent reliability for several bilateral CMJ force-time and strategy-related variables, including peak power, concentric impulse, RSI-modified, and flight time, but weaker reliability for several single-leg and landing-related variables. Advanced CMJ metrics should therefore be treated as decision-support variables, not interchangeable outputs, unless their device-, task-, and variable-specific error is known — a principle consistent with calls for monitoring frameworks built around valid, outcome-specific measures and precise, context-driven decision thresholds (Jimenez and Verhagen, 2025).

### Experimental conditions and applied-setting considerations

The present study was conducted within an applied in-season club environment rather than a fully controlled laboratory setting. This improves ecological relevance for practitioners but also introduces contextual sources of variability. Testing was performed before the main strength and conditioning session, and players maintained their usual team training and match-related routines between sessions. However, training load on the day preceding testing was not experimentally standardized or recorded. Variation in prior-day training exposure, accumulated fatigue, recovery status, sleep, and normal in-season routines may therefore have influenced the athletes’ neuromuscular state at the time of testing.

The task order was also fixed and non-randomized. All players completed bilateral CMJ first, followed by left and right SL-CMJ. This order was chosen to reflect the club’s applied monitoring workflow and to keep the testing sequence identical across participants and sessions. Because VALD ForceDecks, MyJump Lab 3, and Baiobit recorded the same jump trials concurrently, the fixed order is unlikely to explain device differences within a given jump condition. However, fatigue, learning, or potentiation effects across the sequence cannot be excluded, particularly when comparing bilateral and single-leg tasks. The results should therefore be interpreted as estimates from a standardized applied-monitoring protocol rather than from a fully randomized experimental design.

The exploratory SWC analysis further emphasizes the practical consequences of measurement error. SWC values estimated from VALD Session 1 between-athlete SD were approximately 0.6–0.9 cm across jump conditions, whereas MDC_95_ values were substantially larger: 4.11–8.33 cm for device-agreement comparisons and 2.79–8.63 cm for test–retest reliability. Thus, small changes that may be practically relevant in high-performance monitoring may remain below the measurement noise of these device–task combinations.

### Practical implications

In professional club settings, the choice of device should depend on the monitoring purpose and the size of the change that practitioners intend to detect. When precise individual-level monitoring is required, especially when small changes in jump height may influence training or return-to-sport decisions, force-plate testing should be prioritized. In the present study, VALD showed the strongest bilateral CMJ test–retest reliability and the lowest MDC_95_ for bilateral CMJ.

MyJump Lab 3 may be useful as a lower-cost, field-based option for consistent within-device monitoring, provided that the same smartphone, camera setup, rater, protocol, and calculation method are used over time. However, its jump-height output should be interpreted as a flight-time-derived estimate and not as equivalent to VALD impulse–momentum-derived jump height. The small flight-time bias observed in the mechanistic sensitivity analysis suggests that the main discrepancy was related to the height-calculation method rather than gross event-detection error.

Baiobit should be used more cautiously for individual monitoring in this context. Although some mean biases were small, the wider limits of agreement, weaker reliability estimates, and larger MDC_95_ values indicate that small changes may reflect measurement error rather than true neuromuscular change. This caution is particularly relevant for single-leg CMJ, where movement complexity, landing control, and possible sensor–skin motion may amplify measurement variability. If Baiobit is used longitudinally, practitioners should interpret only changes that clearly exceed device-, task-, and metric-specific measurement error.

Overall, outputs from different systems should not be combined within the same athlete profile without method-specific interpretation. The safest applied approach is to use one device, one calculation method, one testing protocol, and athlete-specific baselines, while interpreting changes relative to SEM, MDC_95_, and the practical size of the expected performance change.

## Limitations

This study has limitations. The sample was small, recruited from one professional volleyball club, and restricted to male players, which limits generalizability to other sports, sexes, age groups, and competitive levels. Effective sample sizes also varied across analyses: primary agreement analyses included 12–13 participants and 21–24 participant-session observations, whereas primary test–retest reliability analyses included 10–11 paired athletes depending on the device and jump condition. Several estimates, particularly for single-leg and Baiobit analyses, should therefore be interpreted as precision-limited and require confirmation in larger, preferably multi-club cohorts. Incomplete paired data may have introduced selection bias. Players contributing at least one matched reliability pair were descriptively older and taller than those without paired reliability data, while comparisons of body mass and baseline VALD CMJ performance were limited by sparse data in the non-paired group. The available paired sample may therefore not fully represent the enrolled cohort. Testing was conducted under applied in-season field conditions rather than a fully controlled laboratory design. Prior-day training load was not standardized or recorded, and the task order was fixed and non-randomized. Although all devices recorded the same trials concurrently, training exposure, fatigue, learning, or potentiation effects may have influenced task performance and test–retest reliability estimates. Device-specific limitations should also be considered. Baiobit sensor–skin motion during take-off and landing was not measured directly, despite standardized strap fixation and visual checks before each testing block. MyJump Lab 3 videos were processed by one trained evaluator; inter-rater reliability was not applicable, and intra-rater reliability was not formally assessed because duplicate scoring data were not retained. Finally, the study compared manufacturer-exported outputs rather than raw signals or open algorithms, focused mainly on jump height, and did not perform formal equivalence testing or derive correction equations. The findings should therefore be interpreted as evidence of agreement error and practical non-equivalence under the present testing conditions, not as device-calibration models.

## Conclusions

In male professional volleyball players, smartphone- and IMU-derived jump-height estimates showed meaningful agreement error relative to VALD impulse–momentum-derived jump height. MyJump Lab 3 showed systematic positive bias but comparatively better within-device reliability than Baiobit. Baiobit showed wider agreement error, weaker reliability, and larger MDC_95_ values, particularly in single-leg conditions. Because no *a priori* acceptable agreement threshold was defined and formal equivalence testing was not performed, these findings should be interpreted as evidence of practical non-equivalence rather than as a formal interchangeability decision. Practitioners should avoid combining outputs from different systems within the same athlete monitoring profile and should interpret longitudinal changes using device- and task-specific measurement error, particularly MDC_95_. These conclusions are limited by the small, single-club, male professional volleyball sample and should not be generalized directly to other sports, sexes.

## Data Availability

The datasets presented in this study can be found in online repositories. The names of the repository/repositories and accession number(s) can be found in the article/**Supplementary Material**.
