# Supplementary material for: Agreement and reliability of force-plate, smartphone-based, and wearable-sensor countermovement jump assessments for neuromuscular monitoring in male professional volleyball players
Source: Front Physiol. 2026 Aug 14;17:1902583. doi: 10.3389/fphys.2026.1902583 (PMC13522142; doi:10.3389/fphys.2026.1902583)
Supplement: Supplementary file 1 [file DataSheet1.docx]

**Supplementary material**

**Supplementary Methods**: Analytical refinement from preregistration

The preregistered reliability plan specified SEM = SD × √(1 − ICC) and CV% = SD/mean × 100. In the final analysis, SEM was estimated from the residual error term of the two-way absolute-agreement model, and CV% was calculated as (SD_diff / √2) / grand mean × 100, where SD_diff denotes the standard deviation of paired Session 2 − Session 1 differences. This refinement was adopted because the final reliability analysis focused on paired S1–S2 measurement error and absolute within-athlete change rather than between-participant variability. The same calculation approach was applied consistently across all devices and jump conditions. The change affects the estimation method for absolute error indices but not the primary interpretation of agreement, bias, or limits of agreement. The deviation from the preregistered formula is reported in the supplementary deviation log.

**Supplementary Table S1.** Sensitivity analysis comparing device outputs with VALD flight-time-derived and impulse–momentum-derived jump height.

| **Comparison** | **Jump** | **n** | **Bias (cm)** | **95% LoA lower (cm)** | **95% LoA upper (cm)** | **ICC(2,1)** | **95% CI** | **SEM (cm)** | **MDC₉₅ (cm)** |
| --- | --- | --- | --- | --- | --- | --- | --- | --- | --- |
| MyJump Lab 3 vs VALD flight-time | CMJ | 24 | 0.41 | -2.16 | 2.98 | 0.95 | [0.90, 0.98] | 0.93 | 2.57 |
| MyJump Lab 3 vs VALD flight-time | SL-CMJ-L | 23 | -0.23 | -2.15 | 1.70 | 0.98 | [0.95, 0.99] | 0.69 | 1.93 |
| MyJump Lab 3 vs VALD flight-time | SL-CMJ-R | 22 | 0.09 | -2.24 | 2.41 | 0.90 | [0.78, 0.96] | 0.84 | 2.33 |
| Baiobit vs VALD flight-time | CMJ | 22 | -6.18 | -15.75 | 3.38 | 0.25 | [-0.11, 0.59] | 3.45 | 9.57 |
| Baiobit vs VALD flight-time | SL-CMJ-L | 20 | -2.58 | -8.90 | 3.74 | 0.65 | [0.15, 0.86] | 2.28 | 6.32 |
| Baiobit vs VALD flight-time | SL-CMJ-R | 20 | -1.50 | -7.84 | 4.84 | 0.39 | [-0.01, 0.69] | 2.29 | 6.34 |
| VALD flight-time vs VALD ImpMom | CMJ | 24 | 3.75 | -0.43 | 7.93 | 0.60 | [-0.09, 0.88] | 1.51 | 4.18 |
| VALD flight-time vs VALD ImpMom | SL-CMJ-L | 23 | 2.11 | -1.34 | 5.56 | 0.85 | [0.15, 0.96] | 1.25 | 3.45 |
| VALD flight-time vs VALD ImpMom | SL-CMJ-R | 22 | 2.28 | -1.60 | 6.16 | 0.60 | [-0.04, 0.85] | 1.40 | 3.88 |

*Note. Values are based on the mean of valid trials, with Sessions 1 and 2 pooled at the participant-session level. Bias was calculated as comparator − reference. Positive values indicate higher values for the comparator. For MyJump Lab 3 and Baiobit comparisons, VALD flight-time-derived jump height was used as the reference. The final three rows show the internal VALD comparison between flight-time-derived and impulse–momentum-derived jump height. LoA = limits of agreement; ICC = intraclass correlation coefficient; SEM = standard error of measurement; MDC₉₅ = minimal detectable change at 95% confidence; CMJ = countermovement jump; SL-CMJ = single-leg countermovement jump.*

**Supplementary Table S2.** Exploratory secondary validity analyses: MyJump Lab 3 flight time and Baiobit peak power.

| **Jump type** | **Comparison** | **n** | **Bias** | **95% CI for bias** | **95% LoA** | **ICC(2,1)** | **95% CI for ICC** | **SEM** | **MDC₉₅** |
| --- | --- | --- | --- | --- | --- | --- | --- | --- | --- |
| ***A. Flight time (ms) — MyJump Lab 3 vs VALD*** | | | | | | | | | |
| CMJ | MyJump vs VALD | 24 | 2.59 | [-0.99, 6.17] | [-14.0, 19.2] | 0.96 | [0.91, 0.98] | 5.99 | 16.61 |
| SL-CMJ-L | MyJump vs VALD | 23 | -2.26 | [-6.38, 1.86] | [-20.9, 16.4] | 0.98 | [0.95, 0.99] | 6.74 | 18.68 |
| SL-CMJ-R | MyJump vs VALD | 22 | 0.84 | [-4.36, 6.04] | [-22.2, 23.8] | 0.90 | [0.78, 0.96] | 8.29 | 22.99 |
| ***B. Peak power (W) — Baiobit vs VALD*** | | | | | | | | | |
| CMJ | Baiobit vs VALD | 22 | -2244 | [-2452, -2036] | [-3162, -1325] | 0.03 | [-0.02, 0.14] | 331 | 918 |
| SL-CMJ-L | Baiobit vs VALD | 20 | -1064 | [-1226, -903] | [-1741, -387] | 0.10 | [-0.04, 0.37] | 244 | 677 |
| SL-CMJ-R | Baiobit vs VALD | 21 | -956 | [-1161, -751] | [-1837, -75] | 0.07 | [-0.06, 0.31] | 318 | 881 |

*Note. Bias was calculated as comparator − VALD; positive values indicate higher comparator estimates. Values in Section A are reported in milliseconds and values in Section B in watts. Peak-power analyses are exploratory manufacturer-output comparisons because Baiobit and VALD use non-equivalent algorithms for peak-power estimation. SEM and MDC₉₅ are reported as agreement-error indices for device-comparison analyses. ICC(2,1) = two-way random-effects, absolute-agreement, single-measure intraclass correlation coefficient; LoA = limits of agreement; SEM = standard error of measurement; MDC₉₅ = minimal detectable change at 95% confidence; CMJ = countermovement jump; SL-CMJ = single-leg countermovement jump.*

**Supplementary Table S3.** Summary of secondary test–retest reliability outcomes.

| **Device** | **Outcome** | **ICC(2,1) range** | **MDC₉₅ range** | **CV% range** | **Summary interpretation** |
| --- | --- | --- | --- | --- | --- |
| VALD | Flight time | 0.47–0.90 | 27.71–51.98 ms | 1.7–4.5 | Higher reliability for CMJ and SL-CMJ-L than SL-CMJ-R |
| VALD | Peak power | 0.66–0.96 | 320.72–740.36 W | 2.3–8.9 | Highest reliability observed for CMJ |
| VALD | Concentric impulse | 0.47–0.98 | 9.08–46.63 N·s | 1.3–9.8 | Highest reliability observed for CMJ; lower for SL-CMJ-R |
| VALD | RSI-modified | 0.33–0.95 | 0.05–0.10 m/s | 3.7–15.6 | Higher reliability for CMJ and SL-CMJ-L than SL-CMJ-R |
| VALD | Peak landing force | 0.36–0.60 | 956.82–1897.46 N | 10.3–14.7 | Lower reliability than other VALD force-time outcomes |
| MyJump Lab 3 | Flight time | 0.79–0.95 | 25.92–33.03 ms | 1.6–2.9 | Similar reliability pattern to MyJump jump height |
| Baiobit | RFD | −0.78–0.62 | 4094.96–7811.07 N/s | 25.2–56.7 | Highly variable reliability across jump conditions |
| Baiobit | Reactivity index | −0.59–0.78 | 0.16–0.54 a.u. | 9.4–40.0 | Variable reliability, especially in single-leg conditions |
| Baiobit | Impact ratio | 0.46–0.76 | 0.24–0.30 ratio | 7.4–9.7 | Most consistent Baiobit secondary outcome |

Note. ***This table is intended as a descriptive summary of exploratory secondary reliability analyses; full device-, outcome-, and jump-specific estimates are provided in the OSF repository.*** *Values summarise the range across CMJ, SL-CMJ-L, and SL-CMJ-R conditions. ICC(2,1) = two-way random-effects, absolute-agreement, single-measure intraclass correlation coefficient; MDC₉₅ = minimal detectable change at 95% confidence; CV% = coefficient of variation; CMJ = countermovement jump; SL-CMJ = single-leg countermovement jump; RSI = reactive strength index; RFD = rate of force development; a.u. = arbitrary units.*

**Supplementary Table S4. Participant flow and effective analysis samples**

**Panel A.** Participant and data-availability flow

| Participant/data-flow category | n | Explanation |
| --- | --- | --- |
| Players enrolled | 20 | All players who provided consent and entered the study |
| Players with baseline demographic data available | 19 | Baseline demographic data were unavailable for one enrolled player |
| Players with at least one Session 1 or Session 2 eligible record | 13 | Contributed at least one record eligible for a device–jump analysis |
| Players with at least one matched Session 1–Session 2 reliability pair | 11 | Had paired data for at least one device and jump condition |
| Players with complete matched jump-height records across all nine device–jump combinations | 9 | Had paired data for three devices × three jump conditions |
| Session 2 non-completion related to pain | 1 | Unable to complete the follow-up assessment |
| Session 2 non-attendance, reason undocumented | 2 | Did not attend the follow-up assessment |
| Device-, acquisition-, export-, or QC-specific incomplete records | 4 | Participants could still contribute to analyses for which eligible data were available |

**Panel B.** Effective reliability-analysis samples

| Analysis-sample category | Primary eligible n | Strict-QC n | Explanation |
| --- | --- | --- | --- |
| Players with at least one matched reliability pair | 11 | 11 | Had paired Session 1–Session 2 data for at least one device–jump combination |
| Players with complete matched jump-height records across all nine device–jump combinations | 9 | 8 | Had paired data for three devices × three jump conditions |
| Device- and jump-specific paired reliability samples | 10–11 | 9–11 | Effective paired sample varied according to device and jump condition |

Note. *Eligibility was determined separately for each participant, session, device, jump condition, and outcome. The primary eligible dataset included records retained for the primary analysis, including eligible records with predefined QC flags where applicable. The strict-QC dataset included only eligible records without QC flags. Participants with incomplete records were not excluded from all analyses. No missing observations were imputed. Test–retest reliability analyses required valid matched Session 1 and Session 2 measurements for the relevant device and jump condition.*

**Supplementary Table S5.** Technical details of measurement systems used in the study

| **System** | **Hardware** | **Software/app environment** | **Firmware/iOS information** | **Sampling/video settings** | **Main output used** |
| --- | --- | --- | --- | --- | --- |
| VALD ForceDecks | VALD ForceDecks dual force plates, VALD Performance, Brisbane, Australia | ForceDecks software environment / VALD Hub | Firmware was managed through the ForceDecks software environment; | 1000 Hz native force-time sampling | Impulse–momentum-derived jump height |
| MyJump Lab 3 | iPhone 16, Apple Inc., Cupertino, CA, USA | MyJump Lab 3; internal records described version 3.1 | iOS version not recorded in the available study files | 240 fps video; tripod-mounted 150 cm from the participant and 70 cm above the floor; | Video-derived flight-time jump height |
| Baiobit | Wearable IMU/G-SENSOR 2, Rivelo Srl—BTS Bioengineering Group, Milan, Italy; triaxial accelerometer, triaxial gyroscope, magnetometer, and sensor-fusion processor | Baiobit proprietary software; | Sensor firmware was managed through the Baiobit software environment; | Manufacturer-reported native sensor-fusion output rate: 200 Hz | Manufacturer-exported jump height and secondary metrics |

**Supplementary Table S6. Cluster-bootstrap confidence intervals for Bland–Altman limits of agreement**

| Jump condition | Comparison | n observations / participants | Bias (cm) | Lower LoA (cm) | 95% CI for lower LoA (cm) | Upper LoA (cm) | 95% CI for upper LoA (cm) |
| --- | --- | --- | --- | --- | --- | --- | --- |
| CMJ | MyJump Lab 3 vs VALD | 24 / 13 | +4.16 | −1.24 | −3.08 to +1.42 | +9.56 | +7.98 to +10.64 |
| CMJ | Baiobit vs VALD | 22 / 12 | −2.59 | −10.92 | −13.14 to −6.97 | +5.74 | +2.78 to +7.24 |
| SL-CMJ-L | MyJump Lab 3 vs VALD | 23 / 13 | +1.88 | −2.23 | −3.24 to −0.82 | +6.00 | +3.76 to +7.59 |
| SL-CMJ-L | Baiobit vs VALD | 21 / 12 | −0.46 | −5.83 | −7.19 to −3.96 | +4.90 | +2.38 to +6.65 |
| SL-CMJ-R | MyJump Lab 3 vs VALD | 23 / 13 | +2.36 | −2.16 | −3.56 to −0.20 | +6.89 | +5.02 to +8.22 |
| SL-CMJ-R | Baiobit vs VALD | 21 / 12 | +0.74 | −7.42 | −12.70 to −0.86 | +8.89 | +4.89 to +11.13 |

Note. *Bias was calculated as comparator − VALD. VALD impulse–momentum-derived jump height was the reference. Confidence intervals for limits of agreement were estimated using participant-level cluster bootstrap with 5000 resamples to account for repeated participant-session observations*.

**Supplementary Table S7. Strict-QC primary agreement sensitivity analysis**

| Jump condition | Comparison | n observations / participants | Bias (cm) | 95% LoA (cm) | ICC(2,1) [95% CI] | SEM (cm) | MDC95 (cm) |
| --- | --- | --- | --- | --- | --- | --- | --- |
| CMJ | MyJump Lab 3 vs VALD | 24 / 13 | +4.16 | -1.24 – +9.56 | 0.543 [-0.100, 0.840] | 1.95 | 5.40 |
| CMJ | Baiobit vs VALD | 22 / 12 | -2.59 | -10.92 – +5.73 | 0.490 [0.090, 0.750] | 3.00 | 8.33 |
| SL-CMJ-L | MyJump Lab 3 vs VALD | 23 / 13 | +1.88 | -2.23 – +6.00 | 0.836 [0.360, 0.940] | 1.48 | 4.11 |
| SL-CMJ-L | Baiobit vs VALD | 20 / 12 | -0.49 | -5.99 – +5.01 | 0.810 [0.590, 0.920] | 1.98 | 5.50 |
| SL-CMJ-R | MyJump Lab 3 vs VALD | 23 / 13 | +2.36 | -2.16 – +6.89 | 0.545 [-0.020, 0.820] | 1.63 | 4.53 |
| SL-CMJ-R | Baiobit vs VALD | 21 / 12 | +0.74 | -7.42 – +8.90 | 0.201 [-0.250, 0.580] | 2.94 | 8.16 |

Note. *Strict-QC records were defined as qc_clean_primary only. Bias was calculated as comparator − VALD, using VALD impulse–momentum-derived jump height as the reference. LoA = limits of agreement. ICC(2,1), SEM, and MDC95 were computed using the same agreement functions as the primary validity analysis.*

**Supplementary Table S8. Best-trial primary agreement sensitivity analysis**

| Jump condition | Comparison | n observations / participants | Bias (cm) | 95% LoA (cm) | ICC(2,1) [95% CI] | SEM (cm) | MDC95 (cm) |
| --- | --- | --- | --- | --- | --- | --- | --- |
| CMJ | MyJump Lab 3 vs VALD | 24 / 13 | +5.54 | -0.12 – +11.19 | 0.441 [-0.090, 0.790] | 2.04 | 5.66 |
| CMJ | Baiobit vs VALD | 22 / 12 | +0.51 | -7.84 – +8.86 | 0.598 [0.240, 0.810] | 3.01 | 8.35 |
| SL-CMJ-L | MyJump Lab 3 vs VALD | 23 / 13 | +3.07 | -0.37 – +6.52 | 0.759 [-0.060, 0.940] | 1.24 | 3.45 |
| SL-CMJ-L | Baiobit vs VALD | 21 / 12 | +1.33 | -4.41 – +7.06 | 0.754 [0.470, 0.890] | 2.07 | 5.74 |
| SL-CMJ-R | MyJump Lab 3 vs VALD | 23 / 13 | +3.51 | -0.84 – +7.87 | 0.413 [-0.110, 0.760] | 1.57 | 4.35 |
| SL-CMJ-R | Baiobit vs VALD | 21 / 12 | +1.98 | -6.54 – +10.51 | 0.131 [-0.240, 0.500] | 3.08 | 8.53 |

Note. *Best-trial values used jump_height_cm_best, the predefined maximum valid-trial aggregation in the project dataset. Bias was calculated as comparator − VALD, using VALD impulse–momentum-derived jump height as the reference. LoA = limits of agreement.*

**Supplementary Table S9. Temporal-bias exploratory analysis**

| Session/wave | Jump condition | Comparison | n observations / participants | Mean comparator height (cm) | Mean VALD impulse–momentum height (cm) | Bias (cm) | SD of differences (cm) | 95% LoA (cm) |
| --- | --- | --- | --- | --- | --- | --- | --- | --- |
| Pilot | CMJ | MyJump Lab 3 vs VALD | 13 / 13 | 45.13 | 43.35 | +1.78 | 2.41 | -2.95 – +6.50 |
| Pilot | CMJ | Baiobit vs VALD | 11 / 11 | 40.75 | 43.68 | -2.94 | 3.99 | -10.76 – +4.89 |
| Pilot | SL-CMJ-L | MyJump Lab 3 vs VALD | 11 / 11 | 21.19 | 19.91 | +1.28 | 2.80 | -4.21 – +6.76 |
| Pilot | SL-CMJ-R | MyJump Lab 3 vs VALD | 11 / 11 | 21.27 | 19.65 | +1.61 | 3.22 | -4.69 – +7.92 |
| Pilot | SL-CMJ-R | Baiobit vs VALD | 1 / 1 | 24.80 | 22.70 | +2.10 | NA | NA |
| S1 | CMJ | MyJump Lab 3 vs VALD | 12 / 12 | 43.98 | 40.55 | +3.43 | 2.13 | -0.75 – +7.61 |
| S1 | CMJ | Baiobit vs VALD | 11 / 11 | 38.48 | 40.61 | -2.13 | 3.92 | -9.81 – +5.56 |
| S1 | SL-CMJ-L | MyJump Lab 3 vs VALD | 12 / 12 | 21.04 | 18.89 | +2.15 | 2.29 | -2.34 – +6.65 |
| S1 | SL-CMJ-L | Baiobit vs VALD | 11 / 11 | 19.16 | 19.08 | +0.08 | 2.76 | -5.32 – +5.49 |
| S1 | SL-CMJ-R | MyJump Lab 3 vs VALD | 12 / 12 | 21.37 | 18.76 | +2.61 | 2.81 | -2.91 – +8.12 |
| S1 | SL-CMJ-R | Baiobit vs VALD | 11 / 11 | 19.10 | 18.94 | +0.16 | 5.10 | -9.83 – +10.15 |
| S2 | CMJ | MyJump Lab 3 vs VALD | 12 / 12 | 45.62 | 40.73 | +4.89 | 3.19 | -1.36 – +11.14 |
| S2 | CMJ | Baiobit vs VALD | 11 / 11 | 37.56 | 40.63 | -3.06 | 4.70 | -12.27 – +6.14 |
| S2 | SL-CMJ-L | MyJump Lab 3 vs VALD | 11 / 11 | 21.91 | 20.32 | +1.59 | 1.93 | -2.19 – +5.37 |
| S2 | SL-CMJ-L | Baiobit vs VALD | 9 / 9 | 19.67 | 20.86 | -1.19 | 2.86 | -6.80 – +4.41 |
| S2 | SL-CMJ-R | MyJump Lab 3 vs VALD | 11 / 11 | 21.58 | 19.48 | +2.10 | 1.70 | -1.23 – +5.42 |
| S2 | SL-CMJ-R | Baiobit vs VALD | 10 / 10 | 20.91 | 19.54 | +1.37 | 2.97 | -4.44 – +7.18 |
| S3 | CMJ | Baiobit vs VALD | 9 / 9 | 37.29 | 40.54 | -3.25 | 6.49 | -15.97 – +9.47 |

Note. *Temporal-bias exploratory analyses used qc_clean_primary records and mean jump height. Bias was calculated as comparator − VALD. Pilot and S3 were exploratory waves and were not included in the primary pooled validity analysis. LoA are shown where at least two paired observations were available.*

Figures.

**Supplementary Figure S1.** Participant-level bias profiles relative to VALD impulse–momentum jump height.

Note. *Points show participant-level mean device difference across available paired Session 1 and Session 2 records. Bias was calculated as device − VALD. Grey bands indicate ±VALD MDC₉₅ for each jump condition. Lines connect MyJump Lab 3 and Baiobit estimates from the same participant where both device estimates were available.*

**Supplementary Figure S2.** Exploratory temporal-bias analysis across available testing waves.

Note. *Points represent mean bias calculated as comparator − VALD, using VALD impulse–momentum-derived jump height as the reference. Error bars represent 95% confidence intervals for mean bias. Grey shaded areas indicate exploratory waves. Pilot and S3 were not included in the primary pooled agreement analysis. CMJ = countermovement jump; SL-CMJ = single-leg countermovement jump.*

**Supplementary Figure S3.** Bland–Altman plots for test–retest reliability of jump height.

Note. *Panels show Session 2 − Session 1 differences for VALD, MyJump Lab 3, and Baiobit across bilateral and single-leg CMJ conditions. Solid black lines indicate mean bias, dashed red lines indicate 95% limits of agreement, and grey bands indicate the 95% confidence interval of the mean bias.*

**Supplementary Figure S4.** ICC(2,1) heatmap for secondary test–retest reliability outcomes.

Note. *Cells show ICC(2,1) values for secondary reliability outcomes from Session 1 to Session 2. Values below zero were truncated visually at zero for colour scaling and labelled as <0.00. Colour categories indicate commonly used interpretive thresholds: poor <0.50, moderate 0.50–0.75, good 0.75–0.90, and excellent ≥0.90.*

**Supplementary Figure S5**. Relative-error Bland–Altman plots for primary device-agreement analyses. Percentage difference was calculated as 100 × (comparator − VALD) / [(comparator + VALD)/2], where comparator refers to MyJump Lab 3 or Baiobit. Positive values indicate higher estimates from the comparator device. The solid line represents mean percentage bias, and dashed lines represent the 95% limits of agreement. Points represent participant-session observations; some participants contributed observations from both Session 1 and Session 2. No percentage-error acceptability bands were imposed because no a priori percentage-error threshold was defined.
