# Supplementary material for: Agreement and reliability of force-plate, smartphone-based, and wearable-sensor countermovement jump assessments for neuromuscular monitoring in male professional volleyball players
Source: Front Physiol. 2026 Aug 14;17:1902583. doi: 10.3389/fphys.2026.1902583 (PMC13522142; doi:10.3389/fphys.2026.1902583)
Supplement: Supplementary file 2 [file DataSheet2.docx]

**Completed GRRAS Checklist**

*Guidelines for Reporting Reliability and Agreement Studies*

| **Item** | **GRRAS checklist item** | **Reported in section(s)** | **Where/how addressed in manuscript** |
| --- | --- | --- | --- |
| 1 | Identify in title or abstract that interrater/intrarater reliability or agreement was investigated. | Title; Abstract | Title states 'agreement and measurement error'; Abstract states 'field-based measurement agreement and test-retest reliability study' and describes bias, LoA, ICC, SEM, MDC95. |
| 2 | Name and describe the diagnostic or measurement device of interest explicitly. | Introduction; Methods – Measurement systems | Devices are explicitly named and described: VALD ForceDecks, MyJump Lab 3 on iPhone 16, and Baiobit IMU. Measurement principles and export methods are stated. |
| 3 | Specify the subject population of interest. | Abstract; Methods – Participants | Abstract and Participants section specify elite male volleyball players; n = 20 enrolled; age, body mass, height and Tier 4 classification are reported. |
| 4 | Specify the rater population of interest (if applicable). | Methods – Measurement systems | Manual MyJump processing is relevant because take-off/landing frames were manually identified and 'both raters completed standardised familiarisation and practice scoring'. |
| 5 | Describe what is already known about reliability and agreement and provide a rationale for the study (if applicable). | Introduction | Introduction summarises CMJ use, device proliferation, different acquisition principles, existing validity/reliability evidence, and the unresolved issue of interchangeability under the same field-testing conditions. |
| 6 | Explain how the sample size was chosen. State the determined number of raters, subjects/objects, and replicate observations. | Methods – Sample-size rationale; Participant flow, missing data, and effective analysis samples; Countermovement jump protocol | Sample size rationale is stated as the available population of elite players from one professional club and aligned with exploratory estimation. Number enrolled (20), primary analyzable participants (13), sessions, conditions, and three trials per condition are reported. |
| 7 | Describe the sampling method. | Methods – Participants; Sample-size rationale | Participants were active club players from one professional volleyball club, with eligibility based on active player status and absence of acute musculoskeletal injury at testing. The design is an available/team-based elite sample rather than random sampling. |
| 8 | Describe the measurement/rating process (e.g. time interval between repeated measurements, availability of clinical information, blinding). | Methods – Study design and setting; Measurement systems; Countermovement jump protocol; Data processing and quality control | Testing setting, time of day, acquisition waves, 12-day interval between Session 1 and Session 2, concurrent recording by three systems, warm-up, trial order, rests, instructions, SL-CMJ criteria, repeated trials, data processing, QC, and aggregation are described. |
| 9 | State whether measurements/ratings were conducted independently. | Methods – Study design and setting; Measurement systems | The manuscript states that the three systems recorded the same trials concurrently, but it does not explicitly state whether MyJump raters/processors were independent or blinded to other device outputs. |
| 10 | Describe the statistical analysis. | Methods – Statistical analysis | Statistical analysis is detailed: comparator-minus-reference bias, 95% CI for bias, 95% LoA, ICC(2,1) absolute agreement, SEM, MDC95, CV%, sensitivity analyses, pooled session rationale, and estimation-focused interpretation. |
| 11 | State the actual number of raters and subjects/objects which were included and the number of replicate observations which were conducted. | Results – Data availability and quality control; Tables 1 and 2 | Results report final source dataset (342 participant x session x jump x device rows), 332 eligible records, 309 pass records, 23 flagged records, 13/20 players contributing to primary analyses, and device/jump-specific n in Tables 1 and 2. |
| 12 | Describe the sample characteristics of raters and subjects (e.g. training, experience). | Methods – Participants; Measurement systems | Subject characteristics are reported: age 17.85 ± 0.85 years, body mass 86.09 ± 8.67 kg, height 195.20 ± 5.13 cm, elite male volleyball, Tier 4. Rater familiarisation is reported |
| 13 | Report estimates of reliability and agreement including measures of statistical uncertainty. | Results – Primary agreement: jump height; MyJump flight-time sensitivity; Test–retest reliability; Sensitivity and exploratory analyses; Tables 1 and 2; Supplementary Material | Agreement and reliability estimates are extensively reported: bias, 95% CI for bias in text, 95% LoA, ICC(2,1), SEM, MDC95, CV%, device- and jump-specific tables, Bland-Altman plots, and sensitivity/exploratory results. |
| 14 | Discuss the practical relevance of results. | Discussion; Experimental conditions and applied-setting considerations; Practical implications | Discussion and Practical Implications explain non-interchangeability, device consistency, re-baselining, measurement-error thresholds, MyJump as flight-time-derived monitoring, Baiobit caution, and SL-CMJ interpretation. |
| 15 | Provide detailed results if possible (e.g. online). | Methods – Data processing and quality control; Results; Supplementary Material; Data availability; OSF repository | Manuscript states supplementary material includes 9 supplementary tables and 5 supplementary figures.. OSF is cited for preregistration and archived quality-control audit trails, detailed results, confidence intervals, and participant-level profiles. |
